# Erratum to: Stage 1 registered report: metacognitive asymmetries in visual perception and Stage 2 registered report: metacognitive asymmetries in visual perception

**DOI:** 10.1093/nc/niab046

**Published:** 2021-12-13

**Authors:** Matan Mazor, Rani Moran, Stephen M Fleming

**Affiliations:** Institute of Neurology, Wellcome Centre for Human Neuroimaging, University College London, London WC1N 3BG, UK; Institute of Neurology, Wellcome Centre for Human Neuroimaging, University College London, London WC1N 3BG, UK; Institute of Neurology, Max Planck UCL Centre for Computational Psychiatry and Ageing Research, London WC1B 5EH, UK; Institute of Neurology, Wellcome Centre for Human Neuroimaging, University College London, London WC1N 3BG, UK; Institute of Neurology, Max Planck UCL Centre for Computational Psychiatry and Ageing Research, London WC1B 5EH, UK; Department of Experimental Psychology, University College London, London WC1H 0AP, UK

In the originally published version of this manuscript, the same title—‘Metacognitive Asymmetries in Visual Perception’—was used for both https://doi.org/10.1093/nc/niab005 and https://doi.org/10.1093/nc/niab025 in error.

The title for https://doi.org/10.1093/nc/niab005 should read ‘Stage 1 Registered Report: Metacognitive Asymmetries in Visual Perception’.

The title for https://doi.org/10.1093/nc/niab025 should read ‘Stage 2 Registered Report: Metacognitive Asymmetries in Visual Perception’.

These errors have been corrected. The publisher apologizes for the error.

